# Outcomes of Slow-Absorbable Biosynthetic Mesh (Phasix™) in Hernia Repair at Tertiary Care Center, Riyadh, Saudi Arabia

**DOI:** 10.3390/healthcare14091236

**Published:** 2026-05-04

**Authors:** Ibrahim Al Babtain, Sami Almalki, Wed Alwabel, Bader Alhoumaily, Ghala Albaqami, Shumukh Aldawsari, Khalid Alorf

**Affiliations:** 1 College of Medicine, King Saud bin Abdulaziz University for Health Sciences, Riyadh 14611, Saudi Arabia; 2 General Surgery Department, King Abdulaziz Medical City, Riyadh 14611, Saudi Arabia; 3 King Abdullah International Medical Research Center, Riyadh 14611, Saudi Arabia; 4 Orthopedic Surgery Department, King Abdulaziz Medical City, Riyadh 14611, Saudi Arabia

**Keywords:** hernia recurrence, biosynthetic mesh, outcome, poly-4-hydroxybutyrate, laparoscopic hernia repair, open hernia repair, postoperative complications

## Abstract

**Highlights:**

**What are the main findings?**

- Hernia repair with slow-absorbable biosynthetic mesh (Phasix™) showed a low short-term complication rate and a very low 1-year postoperative recurrence rate (1.3%) across mixed ventral and inguinal hernia types.
- Female sex, prior incisional hernia repair, previous mesh removal, and open surgical approach were independently associated with baseline recurrent hernia.

**What are the implications of the main findings?**

- Slow-absorbable biosynthetic mesh (Phasix™) provides reliable short-term biomechanical support across heterogeneous hernia types and can be safely deployed in routine clinical practice without excess early recurrence or infection.
- Patient- and procedure-related risk factors remain the dominant determinants of recurrence, indicating that optimal outcomes with slow-absorbable biosynthetic mesh (Phasix™) depend on appropriate patient selection and surgical strategy rather than mesh choice alone.

**Abstract:**

Background/Objectives: Mesh repair reduces hernia recurrence compared with suture repair, but permanent synthetic meshes are associated with chronic foreign-body complications. Biosynthetic poly-4-hydroxybutyrate mesh (Phasix™) provides temporary reinforcement with gradual resorption; however, short-term real-world outcomes across different hernia types remain limited. This study evaluated short-term outcomes after Phasix™ hernia repair and identified factors associated with baseline recurrent hernia at presentation. Methods: This retrospective cohort included patients ≥ 16 years who underwent open or laparoscopic hernia repair with Phasix™ at King Abdulaziz Medical City (2020–2023). Patients receiving other mesh types were excluded. Demographic, operative, and postoperative data were analyzed. Multivariable logistic regression identified factors associated with baseline recurrent hernia. Results: Among the 228 patients (51.8% female; mean age 55 years), hernia types were incisional (36.0%), paraumbilical (26.3%), and inguinal (24.6%). Early complications were uncommon: seroma in 2.2% and surgical site infection in 1.8% at 1 month. One-year recurrence occurred in 1.3% (3/228). Female sex, prior incisional hernia repair, previous mesh removal, and open repair independently predicted baseline recurrent hernia, while laparoscopic repair was protective. Conclusions: Phasix™ repair demonstrated low short-term complication rates and rare 1-year recurrence across ventral and inguinal hernias. Short-term outcomes were driven mainly by patient and procedural factors rather than mesh-specific effects.

## 1. Introduction

The concept of employing prosthetic material for mending hernias was first proposed in the late nineteenth century [1]. The primary purpose of using such material is to reinforce compromised tissue while achieving a tension-free repair that integrates with host fibrocollagenous tissue, resulting in lower recurrence rates compared with primary suture repair alone [2,3,4]. As a result, mesh has gained widespread acceptance as the preferred method for addressing ventral and inguinal hernias across open and laparoscopic approaches [2,3].

In the past, sutures were the primary method for repairing incisional and ventral hernias, and non-mesh techniques were also widely used for inguinal hernias. However, various studies have demonstrated that using synthetic mesh for repair significantly reduces recurrence rates compared to relying solely on primary suture repair across both ventral and inguinal hernia surgery [2,3,4]. Despite these advantages, permanent synthetic mesh has been linked to persistent issues like chronic pain—particularly after inguinal (groin) hernia repair—as well as adhesions, fistula formation, and mesh infection [2,5]. In addition, synthetic meshes have been associated with a higher susceptibility to infection compared with biologic materials, with reported infection rates varying widely across clinical series depending on patient risk profile, surgical approach, and degree of contamination [5,6]. Although mesh-related infection is uncommon in clean surgical fields, it remains a high-impact complication because it frequently necessitates reintervention, including mesh explantation, and is associated with increased morbidity and recurrence risks [5]. These risks are further amplified in settings where prior wound infection, enteric contamination, or the presence of a stoma complicate the operative field, as success of mesh repair may be jeopardized by contamination-related failure [6].

To address the limitations associated with permanent synthetic materials, biologic meshes were introduced with the aim of inducing a milder inflammatory response and promoting more organized collagen deposition in comparison; however, randomized clinical trials have not demonstrated superior outcomes, and recurrence may be higher while costs are substantially greater [7]. These limitations have driven interest in alternative materials that provide temporary mechanical support while minimizing long-term foreign-body burden.

A recent advancement in hernia repair involves the utilization of biosynthetic mesh, specifically crafted from poly-4-hydroxybutyrate (P4HB). This material provides initial mechanical strength comparable to traditional polypropylene mesh and undergoes gradual resorption over approximately 12–18 months, eliminating any permanent foreign material from the body [8,9]. However, P4HB mesh (Phasix™) progressively loses tensile strength during degradation, which raises concerns regarding early mechanical weakening and the potential for recurrence in higher-risk repairs. In addition, its effectiveness in mitigating infection risk in complex or potentially contaminated operative fields has not been fully characterized, and real-world data on postoperative complications and short-term outcomes across mixed hernia types and surgical approaches remain limited [8,9].

The aim of this study was to assess the short-term postoperative outcomes and complication rates following hernia repair using slow-absorbable biosynthetic mesh (Phasix™) across different hernia types in open and laparoscopic surgical approaches. A secondary objective was to evaluate factors associated with baseline recurrent hernia at presentation.

## 2. Materials and Methods

### 2.1. Study Design

This study was a retrospective cohort study conducted at King Abdul-Aziz Medical City, Riyadh, Saudi Arabia, between 1 January 2020 and 1 January 2023. The institutional electronic medical record system called ‘BestCare’ was used to collect patient data. Prior to data collection, this research was approved by the Institutional Review Board of King Abdullah International Medical Research Center (No. NRC24R/027/01). The primary objective of this study was to evaluate early postoperative outcomes and complication profiles in patients undergoing hernia repair with slow-absorbable biosynthetic mesh (Phasix™, Becton, Dickinson and Company, Franklin Lakes, NJ, USA, across a range of hernia types and operative approaches. The secondary objective was to analyze factors associated with baseline recurrent hernia at presentation.

### 2.2. Participants

In this retrospective study, demographic and clinical characteristics of patients, types of hernia diagnosed, and postoperative complications following hernia repair at 1, 3, and 6 months and 1 year were retrieved from ‘BestCare’, the institutional electronic medical record system. Inclusion criteria included patients above the age of 16 years old who underwent open or laparoscopic hernia repair using slow-absorbable biosynthetic mesh (Phasix™while exclusion criteria included patients who underwent other hernia repair techniques or received a combination of multiple mesh types. Surgical procedures were further characterized based on operative records, including conversion from laparoscopic to open repair, and mesh placement technique (e.g., onlay and sublay), where documented. A non-probability consecutive sampling technique was used to collect patient data in a standardized confidential form accessible only to the co-authors. The extracted data included independent variables related to demographic, preoperative, intraoperative, and postoperative factors, and dependent variables related to outcomes and follow-up. Hernia recurrence was defined based on clinical assessment and/or imaging findings as documented in the patient’s medical record during follow-up.

### 2.3. Statistical Analysis

The analysis plan for this study included collecting and organizing data using Microsoft Excel. Following data collection, the data were prepared by cleaning, checking for missing values, removing duplicates, and identifying outliers. Consequently, the data were transferred to Statistical Package for Social Sciences (SPSS), version 26.0 (IBM Corp., Armonk, NY, USA), for analysis. The categorical variables were presented as numbers and percentages, while continuous variables were summarized as means and standard deviations. Categorical variables were further compared using the Chi-square test. All tests conducted were considered significant if the *p*-value was less than 0.05. Furthermore, significant results were tested in a multivariate regression analysis to determine the significant associated factors with baseline recurrent hernia at presentation, with corresponding odds ratios and 95% confidence intervals.

## 3. Results

### 3.1. Baseline Patient Characteristics

**Figure 1:**
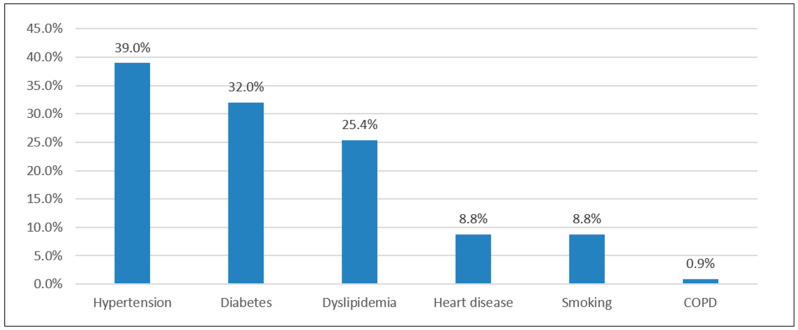
Associated comorbidities.

Baseline patient demographics and clinical characteristics before biosynthetic mesh (Phasix™) hernia repair are presented in Table 1. This study analyzed 228 patients. Slightly more patients were older than 55 years, 115 (50.4%), with more than half being female, 118 (51.8%). The most commonly associated chronic disease was hypertension, 89 (39%), followed by diabetes, 73 (32%) (see also Figure 1). Patients diagnosed with incarcerated hernia constituted 44 individuals (19.3%). Previous incisional hernia was reported in 122 (53.5%) patients, while removal of previous mesh was performed in 10 (4.4%). The most common mesh placement was sublay in 175 (88.4%) patients. Active abdominal wall infections were observed in five (2.2%) patients. A total of 119 (52.2%) patients underwent open surgery directly, and 19 (8.3%) were converted from laparoscopic to open surgery. The mean hernia defect size was 2.56 ± 1.42 cm, with no significant difference between patients with and without recurrence (*p* = 0.835). Using Chi-square testing for these categorical variables, hernia recurrence was more prevalent among female patients (*p* = 0.009), those with previous incisional hernia (*p* < 0.001), those who underwent previous MESH removal (*p* < 0.001), and those who underwent open surgery (*p* = 0.005).

### 3.2. Distribution of Hernia Types

Table 2 shows the distribution of hernia types among the 228 patients. The most common type was incisional hernia, 82 (36%), followed by paraumbilical, 60 (26.3%), and inguinal, 56 (24.6%). Epigastric hernias accounted for 12 patients (5.3%), and other hernia types comprised 18 (7.9%). Hernia type was significantly associated with recurrence on bivariate analysis using the Chi-square test, with differing recurrence patterns observed across hernia types (*p* = 0.008).

### 3.3. Postoperative Complications After Phasix™ Repair

When assessing the complications of patients following hernia repair with slow-absorbable biosynthetic mesh (Phasix™), as illustrated by Table 3, it was observed that there were limited complications across four follow-up periods: 1, 3, and 6 months, and 1 year. The earliest common complication was seroma, which developed in five (2.2%) patients after 1 month, and in only one (0.40%) patient after 3 months. Also, surgical site infection (SSI) cases were seen in four (1.8%) patients 1 month after hernia repair. Other complications were rare, including surgical site occurrence (SSO), defined as any wound complication such as dehiscence or delayed healing not classified as infection or seroma fistula, mesh infection, and bowel perforation, each occurring in separate patients (one each, 4%) during early follow-up. Notably, hernia recurrence was observed in only three (1.3%) patients at the 1-year follow-up. The three recurrence cases included one parastomal hernia and two incisional hernias; one case required conversion from laparoscopic to open repair, one was completed laparoscopically; and one was performed as an open procedure, with mesh placement in sublay (n = 2) and onlay (n = 1) positions. Two of the three patients had a history of prior incisional hernia. No other significant complications were reported beyond the early follow-up periods.

### 3.4. Factors Associated with Hernia Recurrence at Baseline

Multivariable logistic regression analysis, as shown in Table 4, identified factors independently associated with baseline recurrent hernias. Female patients had significantly higher odds of presenting with a recurrent hernia compared to males, with at least 2.5-fold higher odds (AOR = 2.486; 95% CI = 1.190–5.191; *p* = 0.015). Patients with a history of previous incisional hernia surgery were 6.5 times more likely to present with recurrence than those without such history (AOR = 6.537; 95% CI = 2.846–15.014; *p* < 0.001). Similarly, patients who had undergone previous mesh removal had 15.9-fold higher odds of recurrence (AOR = 15.949; 95% CI = 3.206–79.344; *p* = 0.001). In contrast, laparoscopic surgery was associated with lower odds of recurrence compared to open surgery (AOR = 0.369; 95% CI = 0.172–0.791; *p* = 0.010).

## 4. Discussion

### 4.1. Table 1—Patient Characteristics and Factors Associated with Baseline Recurrent Hernia

In this cohort of 228 patients undergoing hernia repair with slow-absorbable biosynthetic mesh (Phasix™), hernia recurrence at baseline was significantly associated with female sex (68.0% vs. 32.0% in males, *p* = 0.009), previous incisional hernia (84.0% vs. 16.0%, *p* < 0.001), previous mesh removal (16.0% vs. 1.1%, *p* < 0.001), and an open surgical approach (68.0% vs. 26.0% laparoscopic, *p* = 0.005). These factors remained independently associated with baseline recurrent hernia on multivariable analysis.

Female sex has been previously identified as a predictor of hernia recurrence. A large systematic review and meta-analysis including over 270 studies of ventral hernia repair reported higher recurrence in female patients compared with males (OR 1.21; 95% CI 1.03–1.42) [10]. Similar sex-based differences have also been reported in inguinal hernia outcomes, potentially related to differences in connective tissue biology and fascial integrity [11].

The strong association between previous incisional hernia and recurrence in our cohort (AOR 6.54) aligns with the prior literature demonstrating that recurrent and incisional hernias carry substantially higher failure rates than primary hernias. Luijendijk et al. reported recurrence rates of up to 48% following repair of recurrent incisional hernias [12], and more recent registry-based studies confirmed that prior hernia repair is one of the strongest predictors of future recurrence in both ventral and inguinal hernia surgery [10,13].

Previous mesh removal showed the highest odds of baseline recurrence (AOR 15.95), reflecting severe underlying tissue compromise. Mesh explantation is typically performed in the context of infection, chronic pain, or mesh failure, all of which are associated with impaired wound healing and distorted anatomy. Large observational studies have shown that mesh removal is associated with markedly increased risk of subsequent hernia recurrence and surgical complexity [14,15].

Surgical approach also demonstrated a significant association with open repair, conferring higher odds of recurrence than laparoscopic repair (AOR 0.37 for laparoscopic). This finding is consistent with contemporary evidence in both ventral and inguinal hernia surgery, where minimally invasive approaches are associated with lower recurrence, reduced wound morbidity, and faster recovery in appropriately selected patients [3,16]. Meta-analyses of inguinal hernia repair have similarly demonstrated lower chronic pain and comparable or lower recurrence with laparoscopic techniques [16].

Although age, smoking status, diabetes, and hypertension were not independently associated with recurrence in our multivariable model, a higher proportion of recurrence was observed among patients with diabetes (42.0% vs. 29.2%), suggesting a biologically plausible trend. Impaired angiogenesis and collagen deposition in diabetes are well-described contributors to wound failure and mesh-related complications [17].

### 4.2. Table 2—Hernia Type and External Validity

Incisional hernias were the most common diagnosis (36.0%), followed by paraumbilical (26.3%) and inguinal hernias (24.6%). Recurrence on bivariate analysis differed by hernia type (*p* = 0.008), with higher recurrence observed in paraumbilical (28.7%) and inguinal hernias (27.0%) compared with epigastric hernias (3.4%).

Importantly, the inclusion of both ventral and inguinal hernias strengthens the external validity of this study by demonstrating outcomes of biosynthetic mesh across heterogeneous hernia phenotypes encountered in routine practice. The mixed hernia spectrum reflects real-world utilization of P4HB mesh rather than selective application in a single anatomic subtype. At the same time, this heterogeneity reflects differences in pathophysiology, operative technique, and recurrence risk across hernia types, which should be considered when interpreting pooled outcomes.

Previous population-based studies confirm that incisional hernias account for approximately 30–40% of abdominal wall hernias encountered in surgical practice [3,10], while inguinal hernias remain the most common hernia worldwide [2]. Recurrence risk varies substantially by hernia type and anatomical location, with incisional hernias demonstrating higher long-term recurrence than primary ventral or inguinal hernias [10,12].

Although hernia type is not included in the multivariable model, the observed bivariate association is clinically meaningful and supports the importance of tailoring surgical strategy and mesh selection to hernia phenotype, defect location, and prior repair history [3,10].

### 4.3. Table 3—Postoperative Outcomes After Phasix™ Repair

Postoperative complications following Phasix™ repair were uncommon across all follow-up intervals. Seroma occurred in 2.2% at 1 month and resolved in most patients by 3 months. Surgical site infection occurred in 1.8% of patients at 1 month and mesh infection was rare (0.4%). Hernia recurrence at 1 year was observed in only three patients (1.3%), involving one parastomal and two incisional hernias.

These low complication rates are consistent with prior reports of P4HB mesh performance. A prospective multicenter study evaluating P4HB mesh in ventral hernia repair reported surgical site infection rates below 5% and recurrence rates under 10% at short-term follow-up [9]. A scoping review of biosynthetic mesh outcomes similarly demonstrated low early recurrence and favorable wound complication profiles compared with permanent synthetic mesh in selected patient populations [8].

The low rate of mesh infection in our cohort is notable, given that mesh infection is one of the most clinically consequential complications after hernia repair and often necessitates mesh explantation [5]. Prior studies have shown that mesh infection is associated with a three- to five-fold increase in recurrence risk following explantation and re-repair [14].

### 4.4. Table 4—Factors Independently Associated with Baseline Recurrent Hernia

Multivariable analysis confirmed female sex, previous incisional hernia repair, previous mesh removal, and open surgical approach as factors independently associated with baseline recurrent hernias, while laparoscopic repair was protective.

These findings are concordant with large meta-analyses of ventral hernia recurrence, which identify recurrent hernia, female sex, and open repair as consistent risk factors [10]. Similar predictors have been reported in long-term registry studies including both ventral and inguinal hernia populations [13,16].

Despite over half of our cohort having high-risk features (51.8% female, 53.5% prior incisional hernia, 52.2% open repair), only 1.3% developed clinical recurrence at 1 year following Phasix™ repair. This suggests that biosynthetic mesh may provide adequate short-term reinforcement even in patients with unfavorable baseline risk profiles, although longer follow-up is required to determine durability beyond the resorption phase [8,9].

### 4.5. Clinical Implications

These findings support the short-term safety and effectiveness of slow-absorbable biosynthetic mesh across mixed ventral and inguinal hernia types in routine clinical practice. The low early recurrence rate observed despite substantial baseline recurrence risk suggests that P4HB mesh (Phasix™) may be a reasonable option in patients at elevated risk for mesh-related complications or in whom permanent foreign material is undesirable.

### 4.6. Limitations and Future Directions

This study is limited by its retrospective design, single-center setting, relatively short follow-up, and absence of a comparator group using alternative mesh types. Hernia recurrence beyond 12 months, particularly after complete resorption of P4HB mesh, could not be evaluated; however, 1-year outcomes remain clinically relevant as they capture early failure and postoperative complication profiles. Selection bias and unmeasured confounders may have influenced surgical approach and mesh placement, as open repair is more likely to be performed in complex or recurrent cases, which inherently carry a higher risk of recurrence. Additionally, the inclusion of multiple hernia types with differing pathophysiologies, surgical approaches, and recurrence risks, and the fact that hernia type was not included in the multivariable model, limited conclusions regarding independent hernia type effects.

Future prospective, multicenter studies with longer follow-up are needed to define the long-term durability of biosynthetic mesh across ventral and inguinal hernia phenotypes and to compare outcomes directly with permanent synthetic and biologic meshes. Also, we recommend recording patient-related factors such as BMI, as well as additional procedural variables—such as mesh overlap, mesh size, primary defect closure versus bridging, fixation methods, and adjunctive techniques (e.g., botulinum toxin A injection, transversus abdominis release, or intraoperative fascia traction)—as these factors may impact recurrence outcomes.

## 5. Conclusions

In this retrospective cohort of 228 patients undergoing hernia repair with slow-absorbable biosynthetic mesh (Phasix™) across different hernia types and surgical approaches, short-term postoperative complication rates were low, and postoperative hernia recurrence at one year was uncommon, with only three patients developing recurrence during follow-up. Although a substantial proportion of patients presented with recurrent hernias at baseline, postoperative recurrence after biosynthetic mesh repair remained minimal. Factors independently associated with baseline recurrent hernia identified in the multivariable model reflected the profile of baseline recurrent hernia in this cohort—female sex, prior incisional hernia surgery, previous mesh removal, and open surgical approach—whereas laparoscopic repair was associated with lower odds of baseline recurrent hernia. Together, these findings indicate that early postoperative recurrence in this cohort was uncommon despite baseline case complexity and support the short-term safety and favorable early performance of biosynthetic mesh in routine clinical practice across diverse hernia types. This paper also contributes region-specific evidence to the limited literature on P4HB mesh performance while highlighting the need for prospective studies with longer follow-up to identify long-term outcomes. These findings should be interpreted in the context of this study’s retrospective, single-center design without a comparator group.

## Figures and Tables

**Table 1:** Baseline patient demographics and clinical characteristics before biosynthetic mesh (Phasix™) hernia repair (n = 228).

Study Variables	Overall N (%) (n = 228)	Hernia Recurrence		*p*-Value ^§^
		No N (%) (n = 178)	Yes N (%) (n = 50)	
Age group				
• ≤55 years	113 (49.6%)	92 (51.7%)	21 (42.0%)	0.226
• >55 years	115 (50.4%)	86 (48.3%)	29 (58.0%)	
Gender				
• Male	110 (48.2%)	94 (52.8%)	16 (32.0%)	0.009 **
• Female	118 (51.8%)	84 (47.2%)	34 (68.0%)	
Associated comorbidities ^†^				
• Diabetes	73 (32.0%)	52 (29.2%)	21 (42.0%)	0.087
• Heart Disease	20 (08.8%)	15 (08.4%)	05 (10.0%)	0.728
• Hypertension	89 (39.0%)	67 (37.6%)	22 (44.0%)	0.415
• Dyslipidemia	58 (25.4%)	43 (24.2%)	15 (30.0%)	0.402
• Smoker	20 (08.8%)	16 (09.0%)	04 (08.0%)	0.827
Incarcerated Hernia				
• No	184 (80.7%)	145 (81.5%)	39 (78.0%)	0.584
• Yes	44 (19.3%)	33 (18.5%)	11 (22.0%)	
Previous Incisional Hernia				
• No	106 (46.5%)	98 (55.1%)	08 (16.0%)	<0.001 **
• Yes	122 (53.5%)	80 (44.9%)	42 (84.0%)	
Previous Mesh Removal				
• No	218 (95.6%)	176 (98.9%)	42 (84.0%)	<0.001 **
• Yes	10 (04.4%)	02 (01.1%)	08 (16.0%)	
Mesh Placement				
• Onlay	23 (11.6%)	16 (10.5%)	07 (15.6%)	0.348
• Sublay	175 (88.4%)	137 (89.5%)	38 (84.4%)	
Active Abdominal Wall Infection				
• No	223 (97.8%)	175 (98.3%)	48 (96.0%)	0.323
• Yes	05 (02.2%)	03 (01.7%)	02 (04.0%)	
Open/Laparoscopic				
• Open	119 (52.2%)	85 (47.8%)	34 (68.0%)	0.005 **
• Lap	101 (44.3%)	88 (49.4%)	13 (26.0%)	
Conversion from Laparoscopic to Open				
• No	209 (91.7%)	163 (91.6%)	46 (92.0%)	0.923
• Yes	19 (08.3%)	15 (08.4%)	04 (0.80%)	
Size of Hernia Defect in cm (mean ± SD)	2.56 ± 1.42	2.54 ± 1.42	2.63 ± 1.47	0.835 ^‡^

^†^ Some patients have more than one comorbidity. ^§^ *p*-value has been calculated using Chi-square test. ^‡^ *p*-value has been calculated using independent sample *t*-test. ** Significant at *p* < 0.05 level.

**Table 2:** Type of hernia diagnosed among patients.

Type of Hernia	Overall N (%) (n = 228)	Hernia Recurrence		*p*-Value ^§^
		No N (%) (n = 178)	Yes N (%) (n = 50)	
Paraumbilical	60 (26.3%)	09 (18.0%)	51 (28.7%)	0.008 **
Incisional	82 (36.0%)	25 (50.0%)	57 (32.0%)	
Inguinal	56 (24.6%)	08 (16.0%)	48 (27.0%)	
Epigastric	12 (05.3%)	06 (12.0%)	06 (03.4%)	
Others	18 (07.9%)	02 (04.0%)	16 (09.0%)	

^§^ *p*-value has been calculated using Chi-square test. ** Significant at *p* < 0.05 level.

**Table 3:** Assessment of complications following hernia repair with slow-absorbable biosynthetic mesh (Phasix™) (n = 228).

Complications	1-Month N (%)	3-Month N (%)	6-Month N (%)	1-Year N (%)
Seroma	5 (2.2%)	1 (0.40%)	0	0
SSI	4 (1.8%)	0	0	0
SSO	1 (0.40%)	0	0	0
Illeus	0	0	0	0
Fistula	1 (0.40%)	0	0	0
Mesh infection	1 (0.40%)	0	0	0
Mesh migration	0	0	0	0
Bowel perforation	1 (0.40%)	1 (0.40%)	0	0
Hernia recurrence	0	0	0	3 (1.3%)

**Table 4:** Multivariate regression analysis to identify factors independently associated with baseline recurrent hernia (n = 228).

**Factor**	**AOR**	**95% CI**	***p*** **-Value**
Gender			
• Male	Ref		
• Female	2.486	1.190–5.191	0.015 **
Previous incisional hernia			
• No	Ref		
• Yes	6.537	2.846–15.014	<0.001 **
Previous mesh removal			
• No	Ref		
• Yes	15.949	3.206–79.344	0.001 **
Open/laparoscopic			
• Open	Ref		
• Lap	0.369	0.172–0.791	0.010 **

Adjusted with age, smoking, diabetes, and hypertension. AOR—adjusted odds ratio; CI—confidence interval. ** Significant at *p* < 0.05 level.

## Data Availability

The data presented in this study are available from the corresponding author upon reasonable request. The data are not publicly available due to institutional and privacy restrictions.

## Abbreviations

The following abbreviations are used in this manuscript:

SSI	Surgical Site Infection
SSO	Surgical Site Occurrence
P4HB	Poly-4-hydroxybutyrate
Phasix™	Poly-4-hydroxybutyrate Biosynthetic Mesh (brand name)
AOR	Adjusted Odds Ratio
CI	Confidence Interval
SD	Standard Deviation
OR	Odds Ratio
Ref	Reference Category (in regression table)
SPSS	Statistical Package for the Social Sciences
IRB	Institutional Review Board
IEHS	International Endohernia Society
HerniaSurge	International HerniaSurge Group (guidelines)
